# Self-administered Vitamin D Status Predictor: Older adults are able to use a self-questionnaire for evaluating their vitamin D status

**DOI:** 10.1371/journal.pone.0186578

**Published:** 2017-11-01

**Authors:** Cedric Annweiler, Anastasiia Kabeshova, Alix Callens, Marie-Liesse Paty, Guillaume T. Duval, Michael F. Holick

**Affiliations:** 1 Department of Neurosciences and Aging, Division of Geriatric Medicine, Angers University Hospital; Angers University Memory Clinic; Research Center on Autonomy and Longevity; UPRES EA 4638, University of Angers, UNAM, Angers, France; 2 Robarts Research Institute, Department of Medical Biophysics, Schulich School of Medicine and Dentistry, the University of Western Ontario, London, Ontario, Canada; 3 Health Faculty, School of Medicine, Angers, France; 4 Section of Endocrinology, Diabetes and Nutrition, Department of Medicine, Boston University Medical Center, Boston, MA, United States of America; University of Alabama at Birmingham, UNITED STATES

## Abstract

**Background:**

The 16-item Vitamin D Status Predictor (VDSP) questionnaire helps to identify, without resorting to a blood test, older adults with low vitamin D concentrations. Our objective was to determine whether a self-administered VDSP was concordant with the VDSP administered by a physician, and to examine the concordance of every single item of the VDSP.

**Methods:**

A total of 349 older in- and outpatients (mean, 83.2±7.2years; 59% female) were consecutively recruited in the geriatric ward of the University Hospital of Angers, France. All participants completed a self-administered VDSP questionnaire (self-VDSP) in paper format composed of 17 items exploring age, gender, general condition, nutrition, vision, mood, cognition, gait and falls, and osteoporosis. All participants underwent subsequently a full clinical examination by a physician exploring the same areas (rater-VDSP).

**Results:**

The agreement between the self-VDSP and the rater-VDSP was almost perfect for the probability of having low vitamin D concentrations, regardless of the definition used (i.e., ≤25, ≤50 or ≤75 nmol/L). The agreements between physicians’ and patients’ responses were significant for every single VDSP item. The agreement was fair to perfect for all items, except for cognitive disorders, undernutrition and polymorbidity (poor agreement).

**Conclusions:**

Older adults are able to evaluate their own probabilities of severe vitamin D deficiency, deficiency and insufficiency. A self-questionnaire may promote the use of the VDSP tool in this population, and help clinicians in decisions to supplement their patients in a reasoned way.

## Introduction

Vitamin D deficiency defined as a 25-hydroxyvitamin D [25(OH)D] <50 nmol/L (20ng/mL) is highly prevalent in children and adults worldwide, where up to 80% are at risk including those who are elderly [1]. Elders with chronic low circulating 25(OH)D concentrations can suffer a significant number of adverse health events including osteomalacia, osteopenia, osteoporosis due to secondary hyperparathyroidism, and non-skeletal consequences with a greater propensity to fall, and increased risks for type 2 diabetes mellitus, cardiovascular disease, deadly cognitive disorders, cancers, sarcopenia and viral upper respiratory tract infections, among others [2].

Importantly, vitamin D deficiency and its consequences may be easily prevented and corrected by oral supplementation and sensible sun exposure [1,3]. For this reason, the use of the 25(OH)D assay, the only reliable indicator for vitamin D status [1], increased dramatically during the last decade. The whole problem is that, although supplementation is relatively cheap, the determination of serum 25(OH)D concentration can cost at least ten times more than a one-year supplementation in some countries [4]. To save heath care costs, US [5] and French [4] health authorities recently examined the clinical utility of the serum testing of 25(OH)D, and concluded that evidence was insufficient to recommend routine vitamin D screening. This recommendation is consistent with the recommendation from the Institute of Medicine [6] and the Endocrine Society’s Practice Guidelines For Vitamin D [7]. As a result, the recommendation is to provide vitamin D supplementation without first measuring the serum 25(OH)D concentration. Consistently, several studies have reported on vitamin D supplementation for up to 6 years without any evidence for toxicity. For instance, Pietras et al. showed that healthy adults receiving 50,000 IU of vitamin D2 twice a month (equivalent to ingesting 3300 IU/day) maintained healthy circulating concentrations of 25(OH)D in the range of 100–150 nmol/L (40–60 ng/mL) [8]. In contrast, there are some recent concerns raised that giving 25(OH)D3 or high doses of vitamin D may increase the risks of falls and allergies among older adults [9,10], which could limit the incentive to supplement without first knowing the vitamin D status. Furthermore, although both population screening and universal supplementation appear justified from a cost-utility point of view in community-dwelling older women and men [11,12], it was reported that population screening was more cost-effective than universal supplementation after the age of 80 years [11], which encourages the use of blood testing in this population.

To avoid the need for a blood test screening for vitamin D status in older adults, we recently developed the Vitamin D Status Predictor (VDSP), a 16-item questionnaire able to identify older adults with undesirable vitamin D status who may be administered vitamin D supplements without obtaining a blood test for 25(OH)D [13,14].

However, the inherent flaw in any rater-administered questionnaire, including the VDSP, relies on the intervention of a caregiver who may influence the results, consumes more resources and can make the test less accessible on a routine basis. The opportunity to answer a self-questionnaire would facilitate its use and potentially extend the possibilities of use in the general population. We hypothesized that the physician-administered VDSP could be self-administered in older adults. The aim of this study was to examine the concordance of answers to a self-administered questionnaire with information collected by a physician during a full clinical examination.

## Materials and methods

### Participants

The study was conducted in accordance with the ethical standards set forth in the Helsinki Declaration (1983). The entire study protocol was approved by the Ethical Committee of the University Hospital of Angers, France (No 2015–03). We studied in- and outpatients aged 65 and over consecutively recruited in the VDSP-G (Vitamin D Status Predictor for Geriatrics) study. The VDSP-G study is an observational cross-sectional study designed to apply the VDSP among all patients consecutively hospitalized or seen in consultation in the geriatric acute care unit of the University Hospital of Angers, France, from March to May 2015 [14]. After giving their written informed consent for research, included participants received a full medical examination consisting of a self-administered VDSP questionnaire, various rater-administered structured questionnaires and a standardized clinical examination.

### Self-administered VDSP questionnaire

A self-administered questionnaire in paper format was given to each patient meeting the selection criteria at their arrival in the ward. This questionnaire consisted of 17 items (Table 1). Except for the age, height, weight, and the number of drugs taken per day, all items corresponded to a question with a forced choice in closed-ended format (i.e., yes or no). If necessary, the self-questionnaire was completed with the help of the relatives. Finally, the algorithms previously published [13] were applied to the items of the self-VDSP to identify among participants those with probable severe vitamin D deficiency (i.e., serum 25(OH)D ≤25nmol/L), vitamin D deficiency (i.e., serum 25(OH)D ≤50nmol/L) or vitamin D insufficiency (i.e., serum 25(OH)D ≤75nmol/L) [1]. Briefly, the VDSP is based on a 16-item questionnaire coupled with combinatorial non-linear algorithms that were built from models of feed forward artificial neural networks (multilayer perceptron) [13,14]. In practice, clinicians submit the 16 responses to the tool, which combines them using three dedicated algorithms to identify severe vitamin D deficiency, deficiency or insufficiency.

**Table 1:** Items of self-assessment by patients.

**Age**	How old are you (in years)? ¦__¦¦__¦¦__¦	Item 1
**Gender**	Are you…? ☐Female or ☐Male	Item 2
**General condition**	Do you think you have a lot of diseases? ☐Yes ☐No Number of different drugs daily taken? ¦__¦¦__¦ Do you live alone? ☐Yes ☐No	Item 13 Item 3 Item 15
**Nutrition**	What is your weight (in kg)? ¦__¦¦__¦¦__¦, ¦__¦ What is your height (in meters)? ¦__¦,¦__¦¦__¦ Do you feel malnourished? ☐Yes ☐No	Item 4 Item 4 Item 12
**Vision**	Do you wear glasses? ☐Yes ☐No	Item 7
**Mood**	Do you regularly take psychoactive drugs? ☐Yes ☐No Do you feel sad? ☐Yes ☐No	Item 6 Item 8
**Cognition**	Do you have memory lapses? ☐Yes ☐No	Item 11
**Gait and falls**	Did you fall in the previous year (at least one fall)? ☐Yes ☐No Do you usually use a walking aid? ☐Yes ☐No Are you afraid of falling? ☐Yes ☐No	Item 10 Item 5 Item 9
**Osteoporosis**	Have you already had vertebral fractures? ☐Yes ☐No Do you usually take osteoporotic medications? ☐Yes ☐No	Item 14 Item 16

#### Medical examination: Rater-administered VDSP questionnaire

Participants underwent a full clinical examination by a physician. The following 16 items from the original VDSP were collected standardizedly: gender, age (in years), number of therapeutic classes used per day, body mass index (BMI, in kg/m^2^), use walking aids, use psychoactive drugs (i.e., benzodiazepines, anti-depressants, and/or neuroleptics), wearing glasses, sad mood, fear of falling, history of falls in the preceding year, cognitive disorders, undernutrition, polymorbidity, history of vertebral fractures, living alone, use osteoporotic medications (bisphosphonates, denosumab, strontium, and/or calcium and vitamin D supplements). The BMI was calculated based on anthropometric measurements. Undernutrition was defined as a BMI below 21 kg/m^2^ [15]. Polymorbidity was defined as having more than three chronic diseases (i.e., diseases of indefinite duration or running a course with minimal change). A fall was defined as an event resulting in a person coming to rest unintentionally on the ground or at other lower level, not as the result of a major intrinsic event or an overwhelming hazard, according to the French Society of Geriatrics and Gerontology (SFGG) and the French national agency for health [16]. The history of vertebral fractures was sought from interview of the patients and/or their relatives and from the medical records. The fear of falling was sought using the following standardized question "Are you afraid of falling?", as previously published [17]. The presence of cognitive disorders was identified using the Mini-Mental State Examination [18] and/or the history of dementia from medical records. Finally, sad mood was sought using the following question from the 4-item Geriatric Depression Scale: "Do you feel discouraged and sad?" [19]. Finally, just as for the self-VDSP, we applied the algorithm previously published [13] to the items of the rater-VDSP to identify among participants those with probable severe vitamin D deficiency or vitamin D deficiency or vitamin D insufficiency [1].

### Statistics

Firstly, physicians’ and patients’ responses regarding patient information were summarized using frequencies and percentages or means ± standard deviations, as appropriate. Secondly, interrater agreement in physicians’ and patients’ quantitative responses was analyzed with the intraclass correlation coefficient (ICC). The ICC is the proportion of the variability in the observations due to the differences between pairs. The ICC takes values from 0 (no agreement) to 1 (perfect agreement) [20]. Finally, agreement in physicians’ and patients’ qualitative responses was calculated with Cohen's kappa (ĸ), which is a coefficient of pairwise agreement between observers [21]. ĸ = 1 implies perfect agreement, and ĸ = 0 suggests that the agreement is no better than that which would be obtained by chance. According to Landis, values are judged on a scale as poor if ĸ≤0.20, fair if 0.21≤ĸ≤0.40, moderate if 0.41≤ĸ≤0.60, substantial if 0.61≤ ĸ≤0.80 and almost perfect if ĸ>0.80 [22]. P-values<0.05 were considered statistically significant. All statistics were performed using SPSS (version19.0; SPSS, Inc., Chicago, IL) and R 3.1.0 (GNU project).

## Results

Table 2 reports the characteristics of 349 included participants (mean±standard deviation, 83.2±7.2years; 59.0% female; 52.7% inpatient; 99.1% Caucasian) obtained with the rater- and self-administered questionnaires. Using the rater-VDSP, 19.5% of participants were classified as having probable severe vitamin D deficiency, 42.2% as having probable vitamin D deficiency, and 78.4% as having probable vitamin D insufficiency. Parallel, using the self-VDSP, 19.2% of participants were classified as having probable severe vitamin D deficiency, 47.9% as having probable vitamin D deficiency, and 85.4% as having probable vitamin D insufficiency.

**Table 2:** Physicians’ and patients’ responses regarding patient information (n = 349).

	Physician responses		Patient responses	
	Summary value	[95% CI]	Summary value	[95% CI]
Item 1- Female gender	206 (59.0)	[53.8–64.2]	202 (57.9)	[52.7–60.1]
Item 2- Age, years (mean±SD)	83.2±7.2	[82.4–84.0]	82.6±8.1	[81.8–83.5]
Item 3- Number of drugs daily taken (mean±SD)	6.0±3.6	[5.6–6.4]	4.8±3.9	[4.4–5.2]
Item 4- Body mass index, kg/m^2^ (mean±SD)	26.0±4.9	[25.5–26.5]	23.8±8.1	[23.0–24.7]
Item 5- Use walking aids	168 (48.1)	[42.9–53.3]	164 (47.0)	[41.8–52.2]
Item 6- Use psychoactive drugs	160 (45.8)	[40.6–51.0]	51 (14.6)	[10.9–18.3]
Item 7- Wearing glasses	191 (54.7)	[49.5–59.9]	205 (58.7)	[53.5–63.9]
Item 8- Sad mood	113 (32.4)	[27.5–37.3]	107 (30.7)	[25.9–35.5]
Item 9- Fear of falling	157 (45.0)	[39.8–50.2]	155 (44.4)	[39.2–49.6]
Item 10- History of falls	174 (49.9)	[44.7–55.2]	147 (42.1)	[36.9–47.3]
Item 11- Cognitive disorders	248 (71.1)	[66.3–75.9]	242 (69.3)	[64.5–74.1]
Item 12- Undernutrition	39 (11.2)	[7.9–14.5]	39 (11.2)	[7.9–14.5]
Item 13- Polymorbidity	207 (59.3)	[54.2–64.5]	34 (9.7)	[6.6–12.8]
Item 14- History of vertebral fractures	17 (4.9)	[2.6–7.2]	27 (7.7)	[4.9–10.5]
Item 15- Living alone	161 (46.1)	[40.9–51.3]	161 (46.1)	[40.9–51.3]
Item 16- Use osteoporotic medications	33 (9.5)	[6.4–12.6]	53 (15.2)	[11.4–19.0]

Summary value presented as n (%) where applicable; CI: confidence interval; SD: standard deviation.

Comparison between rater- and self-VDSP underscored that the agreements between physicians’ and patients’ responses were significant for every single item of the VDSP (Table 3). The P-values were strictly less than 0.001 for all items except for the items 11 to 13 (i.e., cognitive disorders, undernutrition, polymorbidity) with P-values of 0.008, 0.003 and 0.010 respectively. Similarly, the magnitude of the agreement was fair to perfect for all items, with the exception of items 11 to 13 (poor agreement). Specifically, the patients tended to underestimate the existence of cognitive disorders, undernutrition and polymorbidity (Table 2).

**Table 3:** Agreement between physicians’ (rater-VDSP) and patients’ (self-VDSP) responses (n = 349).

	Interrater agreement	[95% CI]	Agreement	P-value
**Quantitative responses**	**Intraclass correlation coefficient**			
Item 2- Age	0.918	[0.898–0.934]	Almost perfect	**<0.001**
Item 3- Number of drugs daily taken	0.769	[0.709–0.817]	Substantial	**<0.001**
Item 4- Body mass index	0.513	[0.380–0.618]	Moderate	**<0.001**
**Qualitative responses**	**Cohen’s Kappa**			
Item 1- Female gender	0.941	[0.906–0.976]	Almost perfect	**<0.001**
Item 5- Use walking aids	0.861	[0.808–0.914]	Almost perfect	**<0.001**
Item 6- Use psychoactive drugs	0.285	[0.202–0.367]	Fair	**<0.001**
Item 7- Wearing glasses	0.813	[0.752–0.874]	Almost perfect	**<0.001**
Item 8- Sad mood	0.579	[0.485–0.673]	Moderate	**<0.001**
Item 9- Fear of falling	0.849	[0.794–0.904]	Almost perfect	**<0.001**
Item 10- History of falls	0.745	[0.674–0.816]	Substantial	**<0.001**
Item 11- Cognitive disorders	0.143	[0.033–0.253]	Poor	**0.008**
Item 12- Undernutrition	0.166	[0.025–0.307]	Poor	**0.003**
Item 13- Polymorbidity	0.072	[0.021–0.123]	Poor	**0.010**
Item 14- History of vertebral fractures	0.383	[0.191–0.575]	Fair	**<0.001**
Item 15- Living alone	1.000	[1.000–1.000]	Perfect	**<0.001**
Item 16- Use osteoporotic medications	0.311	[0.170–0.452]	Fair	**<0.001**
**Vitamin D status classified with the VDSP as:**				
Severe vitamin D deficiency^*^	0.861	[0.772; 0.950]	Almost perfect	**<0.001**
Vitamin D deficiency^†^	0.802	[0.720; 0.884]	Almost perfect	**<0.001**
Vitamin D insufficiency^‡^	0.821	[0.707; 0.935]	Almost perfect	**<0.001**

CI: confidence interval

*: 25(OH)D ≤ 25 nmol/L

†: 25(OH)D ≤ 50 nmol/L

‡: 25(OH)D ≤ 75 nmol/L; P-values indicated as follows: **<0.001** (green); **0.001–0.009** (yellow); **0.010–0.049** (orange); >0.050 (red)

Finally, Table 3 shows that the agreement was almost perfect for the identification of those likely to have severe vitamin D deficiency, vitamin D deficiency or vitamin D insufficiency while using the rater-VDSP or the self-VDSP (Table 3).

## Discussion

The present findings report that the self-administered VDSP shows an almost perfect agreement with the VDSP administered by a physician. The agreement was significant for all separate VDSP items, and the small divergences were mainly linked to the underestimation by patients of their health issues (cognitive disorders, undernutrition, polymorbidity). The clinical relevance is that older adults are able to evaluate their own probabilities of severe vitamin D deficiency, vitamin D deficiency and vitamin D insufficiency, which should save time for caregivers and promote the use of the VDSP tool.

To the best of our knowledge, we report here the first evidence that older adults are able to assess their own probability of hypovitaminosis D, with a high concordance with physician assessment. Only few nonsignificant divergences were observed; all about underreporting by patients of cognitive disorders, undernutrition, polymorbidity. Two explanations may be provided. First, the divergence may result from the use of a question wording in the self-VDSP that was subtly different from the rater-VDSP, and asked patients about their health perception without proposing clear severity level, leaving the patients to judge if they were subject to ‘memory lapses’, ‘malnourishment’ or ‘a lot of chronic diseases’ without specifying a number. Thus, the patients’ answers could vary from the physicians' answers, which were based on a clear definition in the three cases. Second, comparative self-rating and other-rating data may also be considered as “a coin with two faces”: symptom underestimation by the patient can be seen as overestimation by the physician [23]. Third, it should be acknowledged that distorted self-perceptions have already been reported among patients for these three aspects. For instance, subjective memory complaints are not always superimposed with objective cognitive disorders, but may either precede the onset of objective disorders, or be related to anxiety, or be more restrictive than cognitive disorders that may involve cognitive fields other than memory, or be cancelled by anosognosia in the case of dementia [24]. Also, underestimation of weight loss has been regularly reported among patients [25,26]. This general tendency to minimise symptoms is also consistent with the underreporting of polymorbidity here, and has been explained by an overall underestimation of risk perception in patients due to either a lack of knowledge about health issues or a coping strategy [27]. Regardless of the reason for these divergences, their magnitude was not important enough to alter the agreement between the classifications offered by the rater-VDSP and the self-VDSP into individuals with or without severe vitamin D deficiency, deficiency or insufficiency.

Developing new simple, effective and affordable strategies to identify older adults with undesirable vitamin D status is urgent in the context of growing awareness that this condition is common and serious in older adults, with consequent major increases in the number of blood tests and related health costs. Current guidelines regarding the decision to supplement vitamin D in clinical practice are mostly justified by the efficacy of supplementation. For example, the dosage of vitamin D is encouraged in people at high risk of falls and bone fractures [3] because vitamin D supplementation has demonstrated to prevent falls and fractures [28]. This approach, although evidence-based, is yet limited since the question of testing should not be reduced to the efficacy of supplementation. Indeed, despite a relatively low number of clinical trials reporting prevention of adverse health events with vitamin D supplements, accumulating evidence report that vitamin D deficiency precedes and predicts the incident onset of multiple health conditions [2]. Thus, given the limited cost of supplementation and the potential benefits, vitamin D supplementation is desirable among people in whom hypovitaminosis D is observed. This is especially true as it has been calculated that population screening is more cost-effective than universal supplementation after the age of 80 years [11]. However, currently, the only way to diagnose vitamin D deficiency and insufficiency depends on a blood test. That is why, to rationalize the use of serum 25(OH)D assays and save health costs, clinical diagnostic tools like the VDSP are needed to identify individuals at high risk of undesirable vitamin D status, since blood testing continues to remain restrictive due to its expense. The accessibility of such a tool, however, depends on the availability of careers, and it will be improved if the tool is made available directly to the users, who could then test themselves and alert their career in case of probable vitamin D deficiency or insufficiency. This may also open some perspectives in primary care. As older outpatients are able to answer the questionnaire in the waiting room, this approach is easy to implement in clinical practice and will certainly help clinicians in decisions to supplement their patients.

Our results open a new perspective in the field of Quantified Self (QS). QS is a recent trend in the general population based on self-measures of health and function using new digital technologies in order to become healthier or remain healthy [29]. Nowadays, the miniaturization of digital technologies allows measuring human physiological parameters to reflect health status (e.g., blood pressure or caloric expenditures). The main disadvantage of such ‘high-tech’ QS is however to consider the individuals more as measurement objects than actors of their own health, the latter point being yet crucial for health improvement. To promote the active participation of individuals, the World Health Organization recommends using self-administered questionnaires to rate and monitor individuals' health [30–32]. This approach is also thought to educate people about wellness and promote healthy lifestyles [30–32]. Because of the increasing popularity of QS, a self-administered questionnaire evaluating vitamin D status could thus be an interesting solution to identify vitamin D deficiency and insufficiency, to trigger a reasoned strategy of supplementation, and to improve older adults' health.

Besides the originality of the research question on an important issue in clinical routine, the strengths of our study include the standardized collection of data from a single research centre and the testing of older participants of both genders. Additionally, agreements between self-VDSP and rater-VDSP were evaluated using the different consensus definitions of severe vitamin D deficiency, deficiency and insufficiency described in previous literature and used in clinical practice. Regardless, a number of limitations should be acknowledged. First, the study cohort was restricted to in- and outpatients who were probably in poorer health and with more frequent hypovitaminosis D than the population of all seniors. Second, our sample size was relatively small and could not be calculated *a priori*. Third, patients were allowed to get help from their relatives to complete the VDSP, if needed. This aid was authorized as part of the study to be representative of the "real life" where patients have the opportunity to seek for help around them. Fourth, the VDSP was designed to identify older adults with undesirable vitamin D status as defined by low circulating 25(OH)D concentration according to the consensus definition of hypovitaminosis D [1]. Thus, it did not account for other recently discovered D3-hydroxyderivatives such as 20-hydroxyvitamin D_3_, which biological role remains unclear [33]. Finally, although we were able to control for important characteristics that could modify the agreements, other covariables such as the socio-economic conditions or the education level might have influenced the participants' answers to the self-administered questionnaire [34].

In conclusion, our study shows that older adults are able to evaluate their own probability of having severe vitamin D deficiency, deficiency or insufficiency, which should save time for caregivers and promote the use of the VDSP tool. Further research is needed to corroborate this finding by correlating the VDSP tool with serum 25(OH)D levels in people of various ages and health conditions. If confirmed this tool opens new perspective in the approach of evaluating vitamin D deficiency and insufficiency in older adults, and could be useful in daily clinical practice. A VDSP completed by the older adults themselves could provide valuable information to the caregivers, help to identify those with undesirable vitamin D status, and ultimately guide the supplementation plan.
